# Complete Whole-Genome Sequence of *Salmonella enterica* subsp. *enterica* Serovar Java NCTC5706

**DOI:** 10.1128/genomeA.01219-16

**Published:** 2016-11-03

**Authors:** Mohammed-Abbas Fazal, Sarah Alexander, Edward Burnett, Ana Deheer-Graham, Karen Oliver, Nancy Holroyd, Julian Parkhill, Julie E. Russell

**Affiliations:** a Culture Collections, Public Health England, London, United Kingdom; b Wellcome Trust Sanger Institue, Wellcome Trust Genome Campus, Hinxton, United Kingdom

## Abstract

Salmonellae are a significant cause of morbidity and mortality globally. Here, we report the first complete genome sequence for *Salmonella enterica* subsp. *enterica* serovar Java strain NCTC5706. This strain is of historical significance, having been isolated in the pre-antibiotic era and was deposited into the National Collection of Type Cultures in 1939.

## GENOME ANNOUNCEMENT

The salmonellae are major human pathogens that represent a significant global public health issue causing morbidity and mortality resulting in social and economic burden worldwide (1). The genus consists of two species: *Salmonella enterica* and *Salmonella bongori*. There are six subspecies of *S. enterica* differentiated by biochemical tests, namely, subspecies *enterica* (I), *salamae* (II), *arizonae* (IIIa), *diarizonae* (IIIb), *houtenae* (IV), and *indica* (VI) (2). Subspecies I, *S. enterica* subsp. *enterica*, causes 99% of human and animal infections. The two main diseases associated with *S. enterica* are gastroenteritis and typhoidal disease. Here, we report the first complete whole-genome sequence for *Salmonella enterica* subsp. *enterica* serovar Java strain NCTC5706. This strain was deposited in the National Collection of Type Cultures (NCTC) in 1939 by Fritz Kauffmann.

Genomic DNA was extracted using the MasterPure DNA kit and underwent quality controls for high-molecular-weight DNA using the Agilent 2200 TapeStation (>60 kb) and high yield using Qubit (minimum 3 µg DNA). Sequencing was performed on the Pacific Biosciences (PacBio) RS II platform. A 10-kb to 20-kb library was prepared and sequenced using C4/P6 chemistry on single-molecule real-time cells with a 180-min collection protocol on the PacBio RS II. The 10-kb continuous long read was *de novo* assembled using the PacBio hierarchical genome assembly process (HGAP)/Quiver software package, followed by Prokka automated annotation.

A single contig of 4,756,780 bp was generated with a GC content of 52%. The genome did not contain any plasmids. There were 4,376 protein-coding genes, 86 tRNA genes, 181 noncoding RNAs, and 22 rRNA genes organized into seven rRNA operons.

### Accession number(s).

The complete genome sequence has been deposited to the European Nucleotide Archive under accession number SAMEA3468845 within study accession number LT571437.
